# Do we visually estimate intra-operative blood loss better with white or green sponges and is the deviation from the real blood loss clinically acceptable? Results from a simulated scenario study

**DOI:** 10.1371/journal.pone.0240808

**Published:** 2020-10-21

**Authors:** Florian Piekarski, Lara Gerdessen, Elke Schmitt, Linda Tanner, Florian Wunderer, Vanessa Neef, Patrick Meybohm, Kai Zacharowski, Florian Jürgen Raimann

**Affiliations:** 1 Department of Anaesthesiology, Intensive Care Medicine and Pain Therapy, University Hospital Frankfurt, Goethe University, Frankfurt, Germany; 2 Institute of Biostatistics and Mathematical Modelling, Department of Medicine, Goethe University, Frankfurt, Germany; 3 Department of Anaesthesia and Critical Care, University Hospital Würzburg, Wurzburg, Germany; Baylor College of Medicine, UNITED STATES

## Abstract

**Background:**

The intraoperative blood loss is estimated daily in the operating room and is mainly done by visual techniques. Due to local standards, the surgical sponge colours can vary (e.g. white in US, green in Germany). The influence of sponge colour on accuracy of estimation has not been in the focus of research yet.

**Material and methods:**

A blood loss simulation study containing four “bleeding” scenarios each per sponge colour were created by using expired whole blood donation samples. The blood donations were applied to white and green surgical sponges after dilution with full electrolyte solution. Study participants had to estimate the absorbed blood loss in sponges in all scenarios. The difference to the reference blood loss was analysed. Multivariate linear regression analysis was performed to investigate other influence factors such as staff experience and sponge colour.

**Results:**

A total of 53 anaesthesists participated in the study. Visual estimation correlated moderately with reference blood loss in white (Spearman's rho: 0.521; p = 3.748*10^−16^) and green sponges (Spearman's rho: 0.452; p = 4.683*10^−12^). The median visually estimated blood loss was higher in white sponges (250ml IRQ 150–412.5ml) than in green sponges (150ml IQR 100-300ml), compared to reference blood loss (103ml IQR 86–162.8). For both colour types of sponges, major under- and overestimation was observed. The multivariate statistics demonstrates that fabric colours have a significant influence on estimation (p = 3.04*10^−10^), as well as clinician’s qualification level (p = 2.20*10^−10^, p = 1.54*10^−08^) and amount of RBL to be estimated (p < 2*10^−16^).

**Conclusion:**

The deviation of correct blood loss estimation was smaller with white surgical sponges compared to green sponges. In general, deviations were so severe for both types of sponges, that it appears to be advisable to refrain from visually estimating blood loss whenever possible and instead to use other techniques such as e.g. colorimetric estimation.

## Introduction

Blood loss of patients is visually estimated routinely several times every day by clinicians in the operating room [1]. It involves the recording of blood loss in surgical sponges and suction containers, but also recording of external blood loss like surgical clothing or on the floor. As visual blood loss estimation does not require any additional equipment but relies only fully on the availability of experienced clinicians, it is worldwide the prevailing technique to assess blood loss during surgery. However, it is known to be associated with inaccuracies (blood loss under- or overestimation) [2]. Smaller volumes of blood can be visually assessed more accurately than the loss of higher volumes [3–5]. Accounting for this issue, several studies were carried out to quantify its inaccuracy and to improve the visually based method of estimation. Focus laid on practical or web-based training in simulated scenarios [3,6–11]. Several studies investigated visual estimation of blood loss and one study examined the influence of fluid colour on estimation accuracy [12]. But so far, the question if and how sponge colour affects the accuracy of this assessment has not been investigated yet. Depending on local standards, mainly green or white sponges are used in the operative field. Blood in green sponges, unlike as in white ones, tends to show a brown colour instead the signal colour red. Therefore, this could potentially facilitate underestimation in green sponges, whereas white sponges could encourage overestimation. In the present simulation study at a German University hospital, we investigate whether the use of white or green sponges influences the estimation of intra-operative blood volume.

## Material and methods

This study was approved by the Ethics Committee (IRB) at the University Hospital Frankfurt, Goethe University (Ref: 163/19) and was performed in accordance with the Declaration of Helsinki. The data from this single-centre study was collected on 5^th^ of February 2020 at a German university hospital. Participation was voluntary and each participant gave his written consent.

The scope of the study was to evaluate the deviations from the reference in the visual blood loss estimation by anaesthetists caused by the different sponge colours.

### Simulation set-up

Eight simulated bleeding scenarios (four for each of the two sponge colours) were used for evaluation.

For this purpose, expired or unusable due to insufficient filling quantity whole blood donations (provided by the German Red Cross, Institute of Transfusions Medicine, Goethe University Frankfurt, Germany) were mixed with full electrolyte solutions (Sterofundin ISO, B. Braun, Melsungen, Germany) to produce predetermined volumes (77-300ml) with defined haemoglobin (Hb) levels (9.5–12.1 g/dl). This mixture was defined as reference blood loss (RBL). At each step, Hb level was measured by blood gas analysis (Radiometer ABL800 Flex, Radiometer GmbH, Krefeld, Germany). After preparation of the RBL, further full electrolyte solution was added to RBL to simulate typical dilution effects, such as those caused by irrigation, ascites or fluid therapy with crystalloids (4.9–6.2g/dl). This mixture was defined as sample blood loss volume (SBL). To avoid potential confounders, for each scenario, blood donations with the same blood group were used. The blood donations had been treated routinely before with CPD stabilizer solution at the blood donation service to inhibit blood clotting. CPD includes citrate buffer, sodium dihydrogen phosphate, glucose and adenine. Therefore, no other anticoagulant was used in the study.

Two types of surgical sponges were used: white sponges (Curity™ Lap Sponge Sterile, Covidien, Dublin, Irland) and green sponges (FIWA Laparotomy Sponges, Fink u. Walter GmbH, Merchweiler, Germany). The sponges were prepared with a predetermined volume of sample blood. Four different scenarios were created analogously for both, green and white sponges respectively. The amount of sample blood was exactly equal for the corresponding scenarios with either green or white sponges. Scenarios were prepared in a random order. The participants were not informed that one scenario with green sponges and one with white sponges contained the same amount of blood. Each scenario was separated from the other by a partition wall. The participants were anaesthetists with different levels of experience. Study participants were given 1.5 minutes (90 seconds) per scenario to document the visually estimated (V-EBL) total blood loss per scenario in a case report form (CRF). Participants were asked to report the estimated volume per scenario as if they would be in a real situation. In order not to influence the participants, no case details were given in advance. The study was conducted under bright, operating-theatre-like lighting conditions (Median 882 Lux). The lighting conditions were measured with a luxmeter (TFA Dostmann LM37 luxmeter, TFA Dostmann GmbH & Co. KG, Wertheim-Reicholzheim, Germany).

### Statistics

Descriptive statistics for V-EBL with white and green sponges were performed using IBM® SPSS® Statistics (version 26, IBM®, Armonk, New York, USA) and R (version 3.5.1, R Foundation for Statistical Computing, Vienna, Austria). The results are expressed as mean (±SD), median (with interquartile range) or rate (with 95% confidence interval) as appropriate. An additional concordance analysis was performed using the Bland-Altman framework for the agreement between the two measurement methods. Also, Spearman's rank correlation coefficient of VBL (for both sponge colours respectively) and RBL was calculated to compare the performance of V-EBL with respect to RBL in both sponge types. Scatter plots with corresponding univariate (depending only on RBL) linear regression lines were created to illustrate the dependence. Multivariate linear regression analysis was performed to investigate other influence factors such as staff experience (job level and working experience in years) and sponge colour. Results are presented in Table 1. Mann-Whitney and *t*-test was used to compare the difference in the deviations from the reference method of each sponge colour. A p value of < 0.05 was considered to be statistically significant.

**Table 1:** Results of visually blood loss estimation in white and green sponges.

Participants	Statistics	V-EBL in white sponges	Deviations from reference in white sponges (V-EBL—RBL)	V-EBL in green sponges	Deviations from reference in green sponges (V-EBL- RBL)
**All participants**	Median (First Quartil; Third Quartil)	250 (150, 412.5)	133 (33, 283)	150 (100, 300)	33 (-27, 140)
	Mean (± SD)	356.6 ± 313.9	210.9; ± 276.5	219.8 ± 188.8	74.07 ± 175.6
**Senior Consultants**	Median (First Quartil; Third Quartil)	150.0 (100.0, 250.0)	54.83 (-19.5, 135.5)	100.0 (50.0, 177.5)	-27.0 (-67.0, 23.0)
	Mean (± SD)	200.6 ± 146.7	54.8; ± 122.0	126.9 ± 88.8	-18.83 ± 88.6
** Consultants**	Median (First Quartil; Third Quartil)	400.0 (237.5, 500.0)	267 (80.5, 414.0)	400.0 (237.5, 500.0)	100 (23.0, 211.0)
	Mean (± SD)	451.0; ± 334.9	305.3, ± 280.5	451.0 ± 334.9	132.7 ± 173.8
**Trainees**	Median (First Quartil; Third Quartil)	300.0 (172.5, 496.2)	161.0 (70.5, 300.0)	190.0 (100.0, 300.0)	55.0 (-17.0, 161.0)
	Mean (± SD)	388.6 ± 337.1	242.8 ± 298.1	237.8 ± 203.0	92.1 ± 190.7

## Results

### Participants

A total of 53 anaesthesists participated in the study. All 53 CRFs were filled out completely and used for the analysis. The training levels of the anaesthetists were distributed as follows: anaesthesia trainees (52%), consultants (25%) and senior consultants (23%). Median clinical work experience was 3 years for the anaesthesia trainees, 7 years for the consultants and 15 years for the senior consultants.

**Fig 1:**
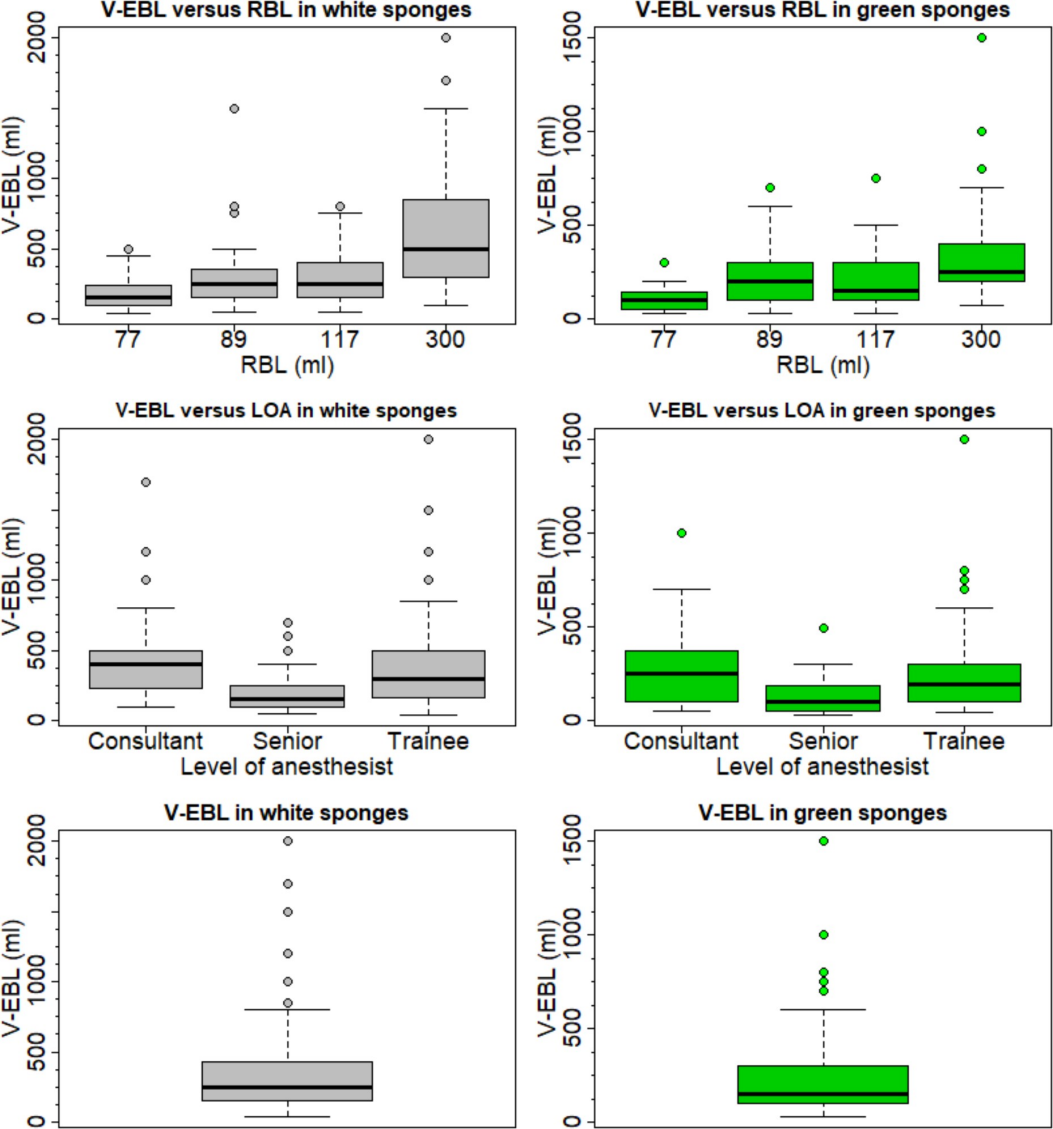
Boxplots for visual blood loss estimation in white and green sponges. Fig 1 demonstrates the visually estimated blood loss (V-EBL) for white and green sponges and the deviation from the reference blood loss (RBL) per scenario, estimates by qualification level (LOA Level of anaesthetist) and total estimates by green and white sponges.

In the descriptive analysis, we were able to show that senior consultants estimated best (V-EBL white or green sponges–RBL), trainees second best and consultants worst for both cloth colours (Table 1). The median estimate of blood loss in white and green sponges also corresponds to this sequence (Table 1). Results of each of the three levels per sponge colour are shown in Fig 1.

Multivariate regression analysis of the estimated volume depending on RBL and doctor’s level was first performed for both of the two subgroups (white and green sponges) separately. In the subgroup multivariate regression analysis, the trainees and consultants showed a significantly different assessment of blood loss in white sponges than the senior consultants (p = 1.29*10^−06^ and p = 1.26*10^−06^, respectively). According to the model, trainees estimate an average of 188.0 ml more than senior physicians, specialists estimate 250.5 ml more than senior physicians. The dependence of the estimates on the RBL is significant (p = 2*10^−16^). We were able to show similar results for green sponges. For example, trainees/ consultants estimate significantly different from senior consultants (p = 0.000102 and p = 9.53*10^−06^), but not significantly different from each other. According to the model, trainees estimate an average of 110.9 ml more than senior consultants, consultants estimate 151.5 ml more than senior consultants. The dependence of the estimates on the RBL is significant (p = 2.14*10^−09^).

### Sponge colour

**Fig 2:**
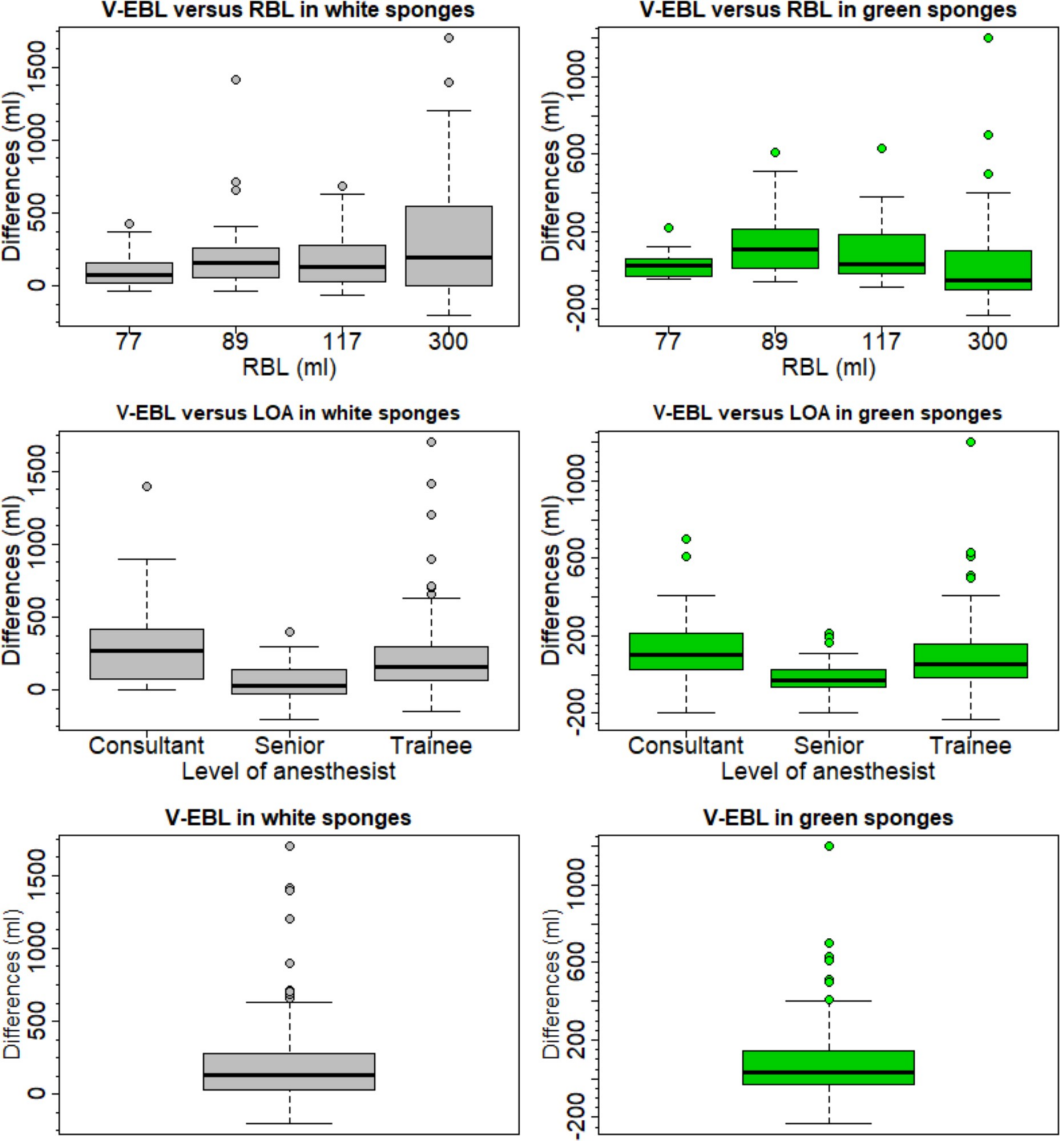
Boxplots for differences between visual blood loss estimation and reference blood loss in white and green sponges. Fig 2 demonstrates the differences between visually estimated blood loss (V-EBL) and reference blood loss (RBL) for white and green sponges per scenario, estimates by qualification level (LOA Level of anaesthetist) and total estimates by green and white sponges.

The difference between the different levels of anaesthesists and the dependence on the real blood loss RBL is not as strong in green sponges as in white sponges but is still highly significant. Overestimation occurred in 86% in white and in 64% in green sponges. Underestimation was more present in green (34%) than in white (16%) sponges. The median visually estimated blood loss was higher in white sponges (250ml IRQ 150–412.5ml) than in green sponges (150ml IQR 100-300ml), compared to RBL (median 103ml, IQR 86–162.8) (Fig 2).

**Fig 3:**
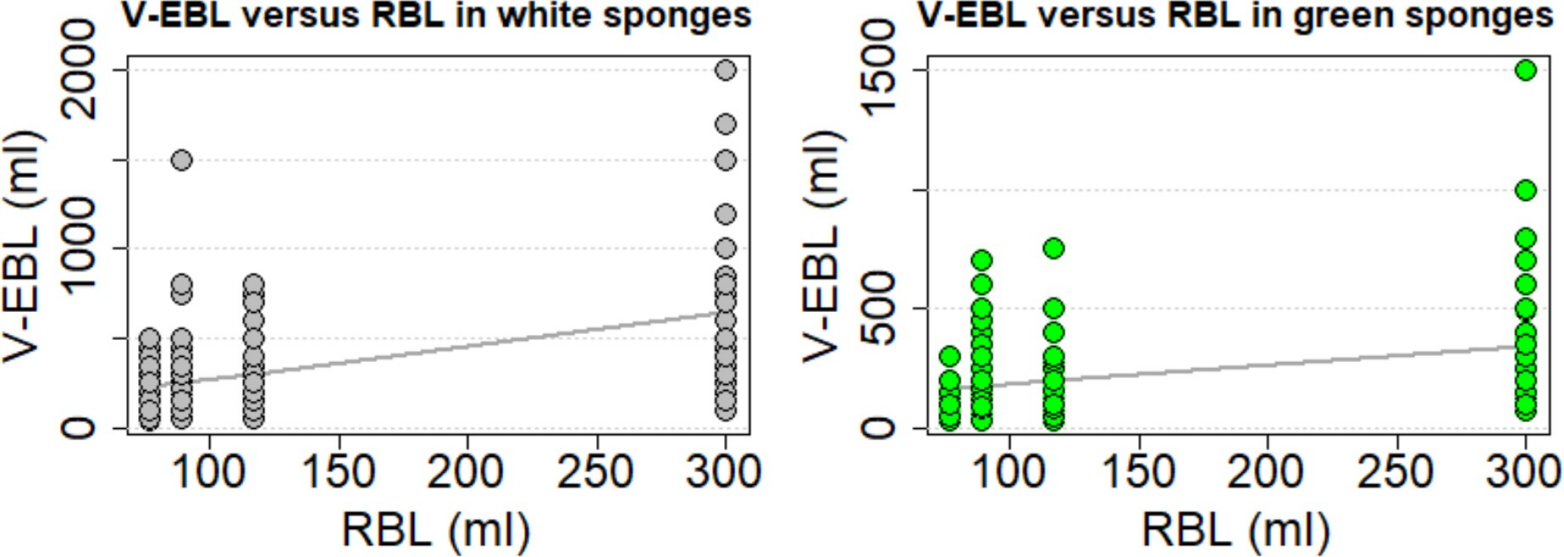
Scatter plots with corresponding regression lines in white and green sponges. Scatter plots for visually estimated blood loss (V-EBL) for white and green sponges and corresponding univariate linear regression line depending on reference blood loss (RBL).

**Fig 4:**
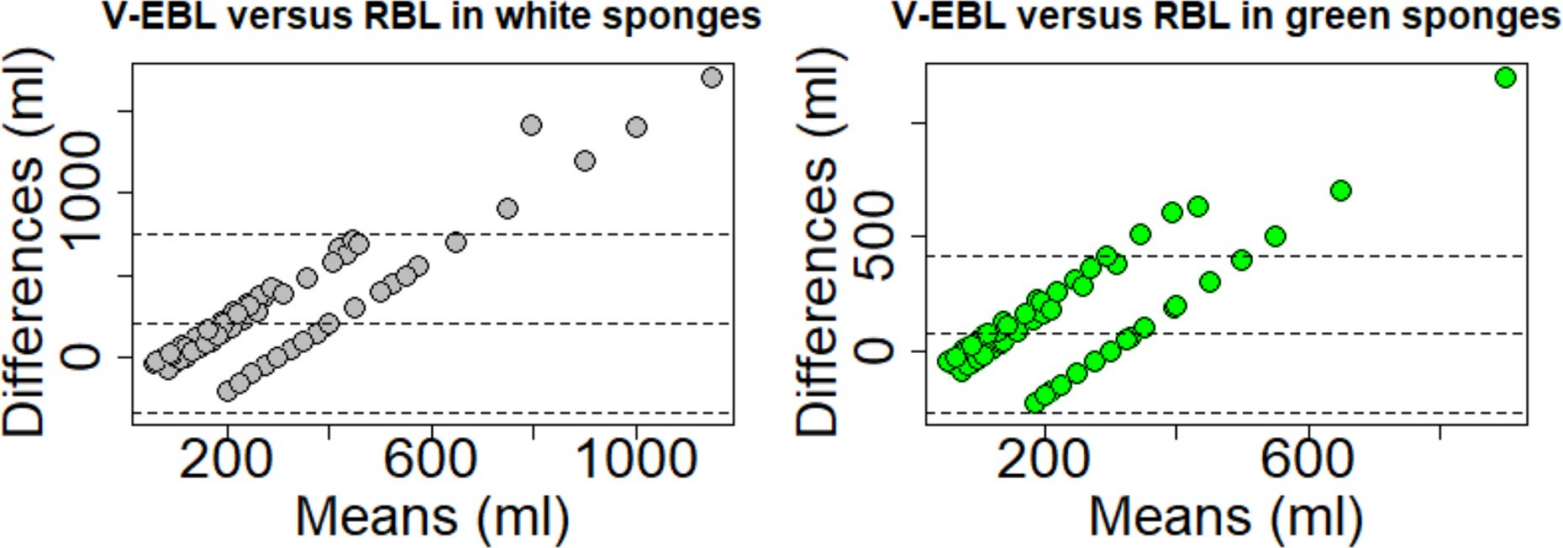
Bland-Altman plots for visually estimated blood loss (V-EBL). Bland-Altman plots for visually estimated blood loss (V-EBL) for white and green sponges compared to reference blood loss (RBL). The Bland Altman plot shows the mean differences (blue line) and agreement interval within 95% of the differences (lower: line; upper line).

**Fig 5:**
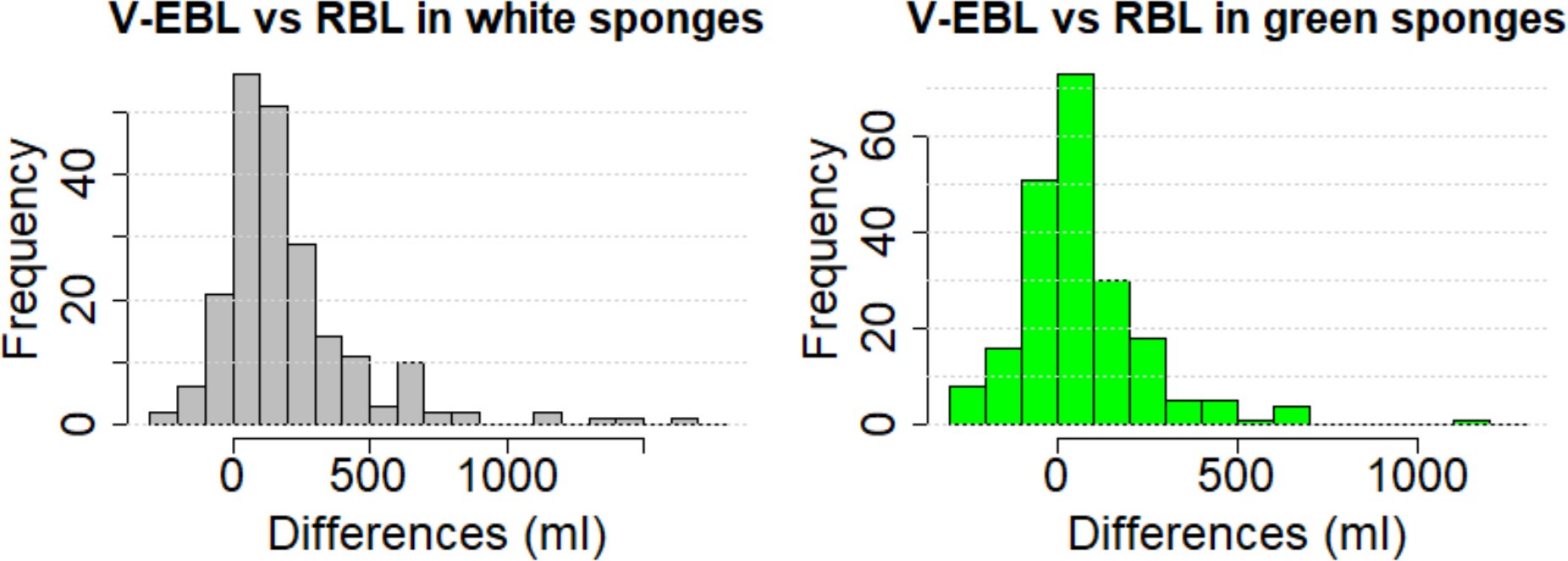
Histograms of differences. Histograms of differences between visually estimated (V-EBL) for white and green sponges and reference blood loss.

The visual estimation for white sponges correlated moderately with reference blood loss (Spearman's rho: 0.521; p = 3.748*10^−16^). Scatter plots with corresponding regression lines illustrate the dependence (Fig 3). Bland Altman plots and histograms of the differences between estimated and reference loss using white sponges are shown in Fig 4. Under- and overestimation was observed in white sponges and ranged from underestimation by 200ml to an overestimation of up to 1700ml (Fig 5).

The visual estimation for green sponges correlated moderately with RBL (Spearman's rho: 0.452; p = 4.683*10^−12^). Bland Altman plots of the differences between V-EBL and RBL using green sponges are shown in Fig 4. Under- and overestimation was observed in green sponges and ranged from underestimation by 230ml to an overestimation of up to 1200ml per station as shown also by additional histograms (Fig 5).

Combined multivariate model estimation of V-EBL depending on the estimates for both green and white sponges, anaesthesist´s level and RBL lead to the following results:

The multivariate statistics demonstrates that fabric colours have a significant influence on estimation (p = 3.04*10^−10^), as well as clinician’s qualification level (p = 2.20*10^−10^, p = 1.54*10^−08^) and amount of RBL to be estimated (p < 2*10^−16^). When using white sponges instead of green ones, in general more blood is estimated (136.8 ml), and again consultants (201.0 ml) and trainees (149.5 ml, here on average for both sponge types) estimate a higher blood loss than senior consultants.

Also results from univariate non-parametric confirmative statistics with paired Wilcoxon test regarding the sponge colour lead to the same conclusion (p = p-value < 2.2*10^−16^ with an estimation of strength of effect by Mann Whitney estimator with 95% CI of 0.66 (0.61–0.71) for white over green sponges) that the sponge colour is significant for visually estimating the blood loss. As pre-tests with shapiro-Wilk test disconfirmed a normal distribution both for green and white sponges’ estimations, no paired t-test but instead the Wilcoxon test was used. In any case, also the paired t-test confirmed a significant difference for the sponge colours (p <2.2*10^−16^).

## Discussion

Our study revealed that the visual measurement of blood loss for both surgical sponge types correlated only moderately with the actual blood loss. In median, estimated blood loss was larger for both white and green sponges compared to the median RBL, whereas in particular for white sponges the overestimation was higher than for green sponges. The presented deviations added up to 1,700 ml per scenario and were therefore severe and of highest clinical relevance. Therefore, we strongly advise against the use of visual blood loss estimation. However, for now V-EBL is the most common method to measure intraoperative blood loss [1].

V-EBL includes estimation of blood loss from surgical sponges and canisters as well as blood on the floor or on surgical clothing. Regardless of professional qualifications, experience or level of qualification, visual estimation is known to be not accurate enough [2–5]. We found relevant differences in the V-EBL depending on the qualification in our simulation. For example, senior consultants estimated better than trainees did, and they again estimated better than consultants did. The results are astonishing, because one would actually assume an increase in experience, so that there is a need for further investigations. A lack of regular feedback on the actual amount of blood loss may be an explanation for the missing learning effect. It has been demonstrated that the visual estimation method can be improved through practical training and tools like pictograms [3,6–11]. In addition to blood collection canisters, surgical sponges play a major role in blood loss estimation, as they absorb blood directly from the surgical field. Intraoperatively, the sponges are gathered by assisting nurses. The colour of the sponges differs among hospitals and countries. So far, there have been no studies on the influence of sponge colour on the estimation accuracy of V-EBL. McConnel et al. investigated the difference between the clinicians estimates for green, red and transparent coloured water on white sponges, but found no difference [12]. In contrast, we were able to show that the use of white sponges leads to more overestimation than the use of green sponges. However, the study was conducted with anaesthesists who are used to green cloths and completely untrained in white, so there is a possible confounding factor. But we also demonstrated in our study that the visual estimation of the participants deviates clinically relevant from the RBL by more than 20%, independent of the colour of the surgical sponge (up to a factor of 16.9 for white and 7.9 for green sponges of RBL). Considering patient blood management (PBM) as a multidisciplinary and evidence-based treatment concept, monitoring blood loss is becoming increasingly important [13,14]. Therefore, the correct quantification of blood loss of greatest importance and demands for viable and reliable alternative methods. In order to obtain more accurate results, further procedures have been evaluated in the past. The weighting of used surgical sponges (gravimetric method) is easy to implement in clinical routine. From the determined net weight (used—unused surgical sponges) the blood volume (conventional conversion factor 1g = 1 ml) is calculated [15–17]. But in the event of greater dilution effects, e.g. large volumes of irrigation, ascites or amniotic fluid, blood loss is here often overestimated.

Instead, calculating the blood loss using different formulas based on laboratory parameters such as Hb level can give an approximation of the bleeding situation [18,19]. However, the formulas assume normovolaemia, so that distortions can occur during a relevant bleeding situation when normovolaemia is not present. Furthermore, the volume effects of intraoperative volume therapy, especially with colloidal fluids or plasma, are not taken into account in these formulas. Therefore, these methods can only serve as a crude approximation in the intraoperative phase. In contrast, the calculation of Hb mass loss is superior to the usual formulas for estimating blood loss, since factors such as dilution have no influence here [20]. However, it must be taken into account that such formulas do not allow real time monitoring of blood loss.

Colorimetric blood loss estimation can provide real-time measurement of blood loss in sponges or canisters. By capturing images of sponges or canisters with a smartphone and using colorimetric image correction the technique is able to calculate the estimated blood loss and loss of Hb mass based on preoperative Hb level [21–24]. Additionally, it provides real-time information on blood loss and potentially improves the treatment of bleeding patients and targeted haemotherapy. So far, colorimetric blood loss estimation is only capable of analysing white sponges [25–29].

### Limitations

This was simulation situation, which means that the usual information and impressions of the operating room are missing. Participants had only a limited time frame at their disposal. Scenarios were only visible from the front, so that a 360° view was not possible. Touching the sponges was also not allowed, due to hygienic precautions. Furthermore, participants in this study were mostly used to work with green sponges and could therefore be unsettled by the use of white sponges. Since the simulated situation is an artificial situation, the Hawthron effect must be taken into account. This effect describes that participants as subjects of a study change their behaviour. Interdisciplinary exchange between surgeons and anaesthesists was lacking, which is normally available during the surgery (e.g. statements by surgeons about extremely heavy bleeding or vascular injuries are included in the considerations of the anaesthesiologist for V-EBL).

## Conclusions

The deviations are on average smaller for white surgical sponges compared to green ones, but in general, deviations are enormous for both sponge types. Therefore, we advise against using visual blood loss estimation, whether in green or white sponges. A more accurate option could be colorimetric blood loss estimation.
